# Laboratory Test Indirectly Reveals the Unreliability of RNA-Dependent 16S rRNA Amplicon Sequences in Detecting the Gut Bacterial Diversity of *Delia antiqua*

**DOI:** 10.3390/insects16060611

**Published:** 2025-06-10

**Authors:** Miaomiao Li, Xin Cao, Linfeng Xu, Luyao Lin, Xiaoqing Wu, Susu Fan, Xinjian Zhang, Fangyuan Zhou

**Affiliations:** Shandong Provincial Key Laboratory of Applied Microbiology, Ecology Institute, Qilu University of Technology (Shandong Academy of Sciences), No. 28789 Jingshidong Road, Licheng District, Jinan 250103, China; 10431221202@stu.qlu.edu.cn (M.L.); 10431221167@stu.qlu.edu.cn (X.C.); 10431230970@stu.qlu.edu.cn (L.X.); 10431230986@stu.qlu.edu.cn (L.L.); xq_wu2008@163.com (X.W.); fansusu1986@hotmail.com (S.F.)

**Keywords:** gut microbe, symbiont, onion maggot, amplicon sequence, insect–microbe, symbiosis

## Abstract

In insect–microbe symbiosis, understanding the diversity of associated bacteria is crucial. DNA-dependent sequence methods are widely used to assess microbial diversity in insects, but they cannot distinguish between live and dead microbes. In contrast, RNA-dependent sequencing can identify alive bacterial communities, making them more suitable for evaluating alive microbiota diversity. However, its practical reliability in insect–microbe symbiosis remains poorly validated. This study investigated larval gut bacteria diversity of *Delia antiqua*, a major pest of Liliaceae crops, by employing both DNA- and RNA-dependent 16S rRNA amplicon sequencing. The reliability of both sequencing methods was evaluated by comparing the effects of synthetic communities (SynComs, constructed according to DNA- or RNA-dependent sequencing) and bacterial communities from wild larvae on axenic larvae. Results revealed significant differences in bacterial community between DNA- and RNA-dependent sequence samples. Compared to bacterial communities from wild larvae, the SynCom constructed based on RNA-dependent sequencing exhibited inhibition effects on *D. antiqua* larvae survival and body weight, while DNA-dependent SynCom did not, suggesting that DNA-dependent methods were superior for assessing symbiotic microbiota in *D. antiqua*. This work will provide insights into microbial diversity detection in *D. antiqua* and offer a framework for other insect–microbe studies.

## Simple Summary

The symbiotic relationship between insects and microorganisms plays a crucial role in the fitness and ecological adaptation of insects. As one of the most threatening pests to Liliaceae crops worldwide, the *Delia antiqua* has been thoroughly studied regarding its biological characteristics and microbial symbionts. We utilized DNA-dependent and RNA-dependent 16S rRNA amplicon sequencing to assess the gut bacterial diversity of *D. antiqua*. The results showed that RNA-dependent sequencing was unreliable for detecting gut bacterial diversity. These findings are of great significance for selecting reliable methods to study the bacterial diversity in the symbiotic relationship between insects and microorganisms.

## 1. Introduction

In the long time of coevolution, microbes and insects have formed tightly symbiotic relationships [1,2,3]. These microbes, including bacteria, fungi, and yeast, inhabit the surface, gut, and intracellular spaces of insects, assisting them in various ways to enhance their fitness [4]. For instance, gut bacteria synthesize diverse vitamins and aid in the digestion of recalcitrant or toxic food [5,6,7,8]. Additionally, symbiotic bacteria influence the growth, development, and social behavior of their insect hosts [6,9]. Those associated microbes, dominated by bacteria, comprise about 10% of their host biomass [1]. All these studies reveal the essential ecological roles of associated bacteria, which in turn have prompted an investigation into insect-associated bacterial diversity.

Currently, the main methods for investigating insect-associated microbial diversity include amplicon sequencing and culture-dependent methods [10]. Amplicon sequencing can provide a comprehensive understanding of the entire microbial community associated with insects. Several sequencing methods have been developed to characterize the diversity of microbial communities [7]. These methods reveal detailed aspects such as the composition, diversity, and structure of bacterial communities in the insect–microbe symbiosis [6,8]. The commonly used DNA-dependent detection method offers high accuracy due to the DNA’s stability, but cannot distinguish between live and inactive bacteria, limiting the understanding of microbiome functions and interactions [11]. Emerging RNA-dependent sequencing can detect active bacteria [12]. RNA-dependent methods are, thus, more suitable for assessing the diversity and function of working microbiota [13,14]. The conserved nature of the 16S rRNA gene makes it a primary target for an amplicon-dependent analysis of the microbial communities, using both RNA (cDNA) and DNA as templates for sequencing of the 16S rRNA gene to infer microbial vitality and metabolic activity [11].

As one of the most threatening pests of *Liliaceae* crops worldwide, *Delia antiqua*, i.e., onion maggot, has been well investigated regarding their biological and ecological characteristics [15]. Based on the axenic *D. antiqua* obtained in the lab [16], the ecological roles of microbes associated with *D. antiqua*, especially their roles in inhibiting entomopathogen infection [17,18] and increasing pesticide resistance [6,19,20,21] have been investigated. All those studies indicate that *D. antiqua* forms a close symbiotic relationship with its associated microbiota. Although several works, using culture-dependent and -independent methods, have detected the diversity of the bacterial community associated with *D. antiqua* [19,22], no experimental comparisons were conducted to evaluate the reliability of RNA- and DNA-dependent 16S rRNA amplicon sequences. Thus, this study compared the diversity of the gut bacterial community associated with *D. antiqua* using DNA- and RNA-dependent sequencing methods, and the gut bacteria of *D. antiqua* were isolated and identified. In addition, the reliability of both sequencing methods was evaluated by comparing the effects of synthetic communities (SynComs, constructed according to DNA- and RNA-dependent sequencing) and bacterial communities from wild *D. antiqua* on larvae. This study not only enriches our knowledge of microbial diversity associated with *D. antiqua,* but also provides a theoretical basis for exploring associated microbial diversity in insect–microbe symbiosis.

## 2. Materials and Methods

### 2.1. Insect Rearing and Microbial Culture Media

Adults of *D. antiqua* were originally collected from garlic fields located in Fan Town, Tai’an City, Shandong Province, China (N 36°14′, E 117°25′) in 2024. Those insects have been reared since then for generations in the laboratory according to methods previously reported [18]. Specifically, the adults *D. antiqua* were kept in insect-rearing cages (45 cm × 45 cm × 45 cm) within an incubator (16L:8D, RH:45%, 20.0 °C). The larvae were reared in 90 mm Petri dishes and were fed on fresh scallion stems. For this experiment, 3rd instar larvae collected from the same Petri dishes were used to prepare the samples for microbial diversity investigation and bacterial isolation. Three types of media, including TSA (OXOID, CM0131B, Basingstoke, UK), R2A (OXOID, CM0906B, Basingstoke, UK), and NA (OXOID, CM1160B, Basingstoke, UK), were used to isolate bacteria from the larval gut.

### 2.2. Larval Gut Sample Collection

Thirty-six 3rd instar larvae of *D. antiqua* from the same Petri dish were randomly collected. Those larvae were then dissected according to methods previously reported [18]. Gut samples were randomly assigned to DNA and RNA groups, with each group containing 18 larvae (Each group consisted of 6 replicates, and each replicate contained guts from 3 larvae). Samples of DNA and RNA groups were used for DNA- and RNA-dependent 16S rRNA amplicon sequences, respectively. Additionally, another 30 3rd instar larvae, collected from the same Petri dish as the above larvae, were dissected, and the gut samples were used for the isolation of gut bacteria.

### 2.3. DNA- and RNA-Dependent 16S rRNA Amplicon Sequence

#### DNA/RNA Extraction and Amplicon Sequencing

Total DNA was extracted from *D. antiqua* larval gut samples using the E.Z.N.A.^®^ soil DNA Kit (Omega D5656-01 Bio-Tek, Norcross, GA, USA) following the manufacturer’s protocol. Total RNA was extracted from the larval gut samples using the QIAsymphony RNA Kit (Qiagen 931636, Hilden, Germany) according to the manufacturer’s instructions and reverse transcribed into cDNA using the QuantiTect Rev. Transcription Kit (Qiagen, 205311, Hilden, Germany). The DNA and cDNA samples, i.e., DNA-dependent sequence samples and RNA-dependent sequence samples, were assessed for quality on a 1% agarose gel, and their concentration and purity were determined using a NanoDrop 2000 UV-vis spectrophotometer (Thermo Scientific, Wilmington, NC, USA). The V3–V4 region of the bacterial 16S rRNA gene was amplified. Purified PCR products were pooled in equimolar ratios and subjected to paired-end sequencing on an Illumina MiSeq PE300 platform (Illumina, San Diego, CA, USA). Detailed information regarding DNA/RNA-dependent amplicon sequencing was provided in the Supplementary Methods.

The data were analyzed through the free online platform of the Majorbio I-Sanger cloud platform (www.i-sanger.com accessed on 2 May 2025) using QIIME 2.0 [23]. Specifically, alpha diversity indices were calculated with the ‘alpha_diversity.py’ script, and compared between DNA and RNA groups using Mann–Whitney test. Non-metric multidimensional scaling (NMDS) and analysis of similarities (Adonis) were used to analyze sample clusters and composition differences. A phylogenetic tree combined with sequence abundance data was used for the weighted Unifrac principal coordinate analysis (PCoA). PERMANOVA based on the weighted UniFrac distance was used to determine community composition differences. The Mann–Whitney test was used to compare genus abundance between the DNA and RNA groups. Detailed information regarding sequence data analysis was provided in the Supplementary Methods.

### 2.4. Isolation and Identification of D. antiqua Gut Bacteria

Three culture media, including TSA (OXOID, CM0131B, Basingstoke, UK), R2A (OXOID, CM0906B, Basingstoke, UK), and NA (OXOID, CM1160B, Basingstoke, UK), were used for the isolation of *D. antiqua* larval gut bacteria according to previous methods with minor revisions [22,24]. Specifically, gut samples from 3rd instar larvae were placed into a 1.5 mL centrifuge tube. The samples were then homogenized, and 1 mL of phosphate-buffered saline (PBS) was added to dilute the solution to a range of 10^−2^ to 10^−9^. Those solutions were then spread on three types of culture media plates. Three days later, individual colonies on Petri dishes (10^−5^) were selected and purified with two rounds of streaking. Those bacterial strains were then transferred to 30 mm culture media plates for further identification.

Bacterial strains were identified by 16S rDNA as described previously [20]. Specifically, the total bacterial DNA was extracted with a PureLink™ Microbiome DNA Purification Kit (Thermo Scientific, A29790, Waltham, MA, USA), and the 16S rDNA gene was amplified with 1492 R and 8F primers. The PCR reactions were conducted as follows: 5 min at 94 °C; 40 cycles of 30 s at 94 °C, 30 s at 51 °C, and 1.5 min at 72 °C; with an extension for 10 min at 72 °C. The PCR products were sequenced by an ABI 3730XL DNA analyzer (Applied Biosystems, A41046, Foster City, CA, USA) with the primers 1492R and 8F. Sequences were manually assembled and edited with MEGA X [25].

All obtained 16S rDNA sequences were aligned online with the BLAST search (http://blast.ncbi.nlm.nih.gov/Blast.cgi accessed on 21 December 2024), and the sequences of the closest strains were downloaded for phylogenetic analysis. *Anabaena affinis* (AF247591) was selected as an outgroup. The phylogenetic tree was constructed with MEGA X (Tamura-Nei model, Maximum Likelihood method, 1000 bootstrap). Subsequently, the phylogenetic tree was visualized and edited in TreeGraph 2 [26] and Photoshop CS5.

### 2.5. SynComs Constructed According to DNA/RNA-Dependent Amplicon Sequencing

To assess the reliability of the DNA- and RNA-dependent amplicon sequencing in detecting the intestinal bacterial diversity of *D. antiqua*, two synthetic communities (SynComs) were constructed with bacterial strains isolated from *D. antiqua* larvae.

For the RNA community, suspension of the above isolated bacterial strains was mixed with the following proportion: LC621:LC623:LC632:LC644:LC645 = 16:1:1:1:1. For the DNA community, suspension of the above bacterial strains was mixed with the following proportion: LC621:LC623:LC632:LC644:LC645 = 60:15:20:1:4. Among them, strains used in both the DNA and RNA groups were first cultured in TSB liquid medium with shaking, and then prepared into individual bacterial suspensions at a concentration of 10^7^ CFU/mL using PBS.

### 2.6. Effects of SynComs on Larval Survival and Growth

Two synthetic communities (SynComs) were constructed with bacterial strains isolated from *D. antiqua* larvae, and their effects on larval growth and survival were compared with the microbial community from laboratory-reared non-axenic larvae.

The bacterial load where the laboratory-reared non-axenic larvae were reared and the wild larvae inhabited (the rhizosphere soil of garlic) was first quantified. For the laboratory-reared non-axenic larvae, as described above, all contents, such as rotted scallion stems in one Petri dish (90 mm) in which the larvae were reared, were collected (more than 50 larvae inside). Subsequently, those contents were resuspended with 10 mL of PBS. Bacterial cell counts in the above suspension were quantified by spreading them on TSA plates, and the bacterial cell counts in the garlic rhizosphere were quantified similarly. There were about 9.7 × 10^7^ CFU/g bacteria in the content inside the Petri dish mentioned above, and about 2.6 × 10^7^ CFU/g bacteria in the garlic rhizosphere soils. Thus, the bacterial cell suspension dose of 10^7^ CFU/mL was used as the final concentration to re-inoculate SynComs into sterilized eggs.

SynComs constructed with dominant bacterial genera as well as the microbial community from the laboratory-reared non-axenic larvae were re-inoculated into sterilized eggs to compare their effects on *D. antiqua* larval survival and weight. Synthetic communities (SynComs) were constructed based on the abundances obtained from the DNA and RNA sequencing. Specifically, *D. antiqua* eggs were sterilized as described previously [21]. In total, 100 sterilized eggs were transferred into a 90 mm Petri dish containing a piece of sterilized filter paper. Those eggs were further inoculated with 1 mL of bacterial cell suspension corresponding to DNA, RNA, and Wild microbial communities individually. To prepare bacterial cell suspensions corresponding to those groups, bacteria were separately cultured with shaking in TSB (OXOID, CM0129B, Basingstoke, UK). SynCom inocula were standardized to 10^7^ CFU/mL, mirroring natural bacterial loads in wild larvae, to ensure ecological relevance. For the wild community, the bacterial suspension derived from rotted scallion stems in Petri dishes (90 mm) containing the larvae mentioned above was used (10^7^ CFU/mL). In addition, axenic larvae [16] were used as the control group (CK). To prevent further propagation, all four groups of bacterial suspension were prepared with PBS, and no other nutrients were added to the Petri dishes containing the sterilized eggs. Four days later, the egg hatch was checked. Two days later, the hatched larvae were transferred into another Petri dish and reared with artificial diets [21]. Another 10 days later, larval survival and body weight were determined as described previously [21]. An individual Petri dish was regarded as one replicate, and 5 replicates were set for each treatment.

At the end of the experiment, three surviving larvae were randomly selected from each replicate of the DNA and RNA groups. Those larvae were dissected to collect the larval gut samples. Gut samples from the same Petri dish were mixed and homogenized for further bacterial isolation and identification. Isolation frequency for each bacterial species was calculated as described above to detect the gut bacterial composition of the surviving larvae.

### 2.7. Data Analysis

Egg hatch percent, larval survival, and larval body weight were compared with one-way ANOVA followed by Duncan’s multiple tests.

## 3. Results

### 3.1. Significant Differences in α-Diversity and β-Diversity of Bacterial Communities Between DNA- and RNA-Dependent Sequence Samples

**Figure 1:**
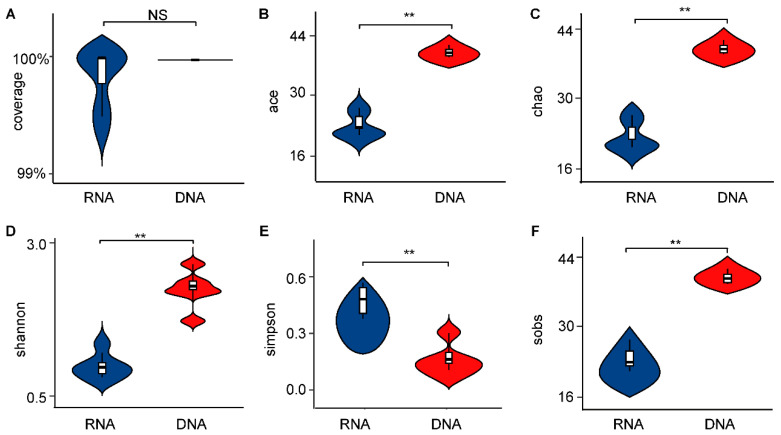
The α index of the intestinal bacterial microbiota in *D. antiqua* larvae was determined using DNA- and RNA-dependent sequence methods. (**A**) coverage, (**B**) Ace index, (**C**) Chao index, (**D**) Shannon index, (**E**) Simpson index, (**F**) Sobs index. The DNA group refers to the group tested using a DNA-dependent sequence, while the RNA group refers to the group tested using cDNA. The “**” above the connected violin plots in the figures indicates a significant difference between the connected plots as determined by a Mann–Whitney test, while "NS" indicates no significant difference. (*p* < 0.05).

In total, 863,120 sequences were generated from larval gut samples, and those sequences were assigned to 1241 ASVs at a 97% similarity cutoff level. The rarefaction curves of the samples were almost flat, indicating the high quality of the data in this experiment and ensuring the reliability of subsequent analyses (Supplementary Figure S1). The Mann–Whitney test was used to compare the α-diversity indices (Ace, Chao, coverage, Shannon, Sobs, and Simpson index). Although there were differences between the two groups for the coverage (Figure 1A, Mann–Whitney test, U = 14.0, *p* = 0.1757), the coverage of both groups was higher than 99.99%. Furthermore, significant differences between the DNA- and RNA-dependent sequence samples were detected for α-diversity indices. Specifically, the Ace (Figure 1B, Mann–Whitney test, U = 0.0, *p* < 0.01), Chao (Figure 1C, Mann–Whitney test, U = 0.0, *p* < 0.01), Shannon (Figure 1D, Mann–Whitney test, U = 0.0, *p* < 0.01), and Sobs (Figure 1F, Mann–Whitney test, U = 0.0, *p* < 0.01) indices for the DNA-dependent sequence group were significantly higher than those of the RNA-dependent sequence group. Considering the evenness of community species, the community diversity of the RNA group was greater than that of the DNA group, as indicated by its higher Simpson index (Figure 1E, Mann–Whitney test, U = 36.0, *p* < 0.01).

**Figure 2:**
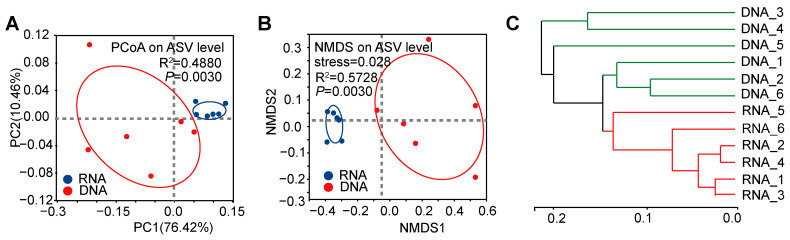
The β-diversity of the intestinal bacterial microbiota in *D. antiqua* larvae was determined using DNA- and RNA-dependent sequence methods. PCoA and NMDS plots for intestinal bacterial microbiota from *D. antiqua* larvae were shown in (**A**) and (**B**), respectively. A hierarchical clustering tree was constructed on the ASV level for the intestinal bacterial microbiota obtained from *D. antiqua* larvae (**C**). The DNA group represents samples tested using DNA-dependent sequence, while the RNA group represents those tested using cDNA. The PCoA plots were generated based on the weighted UniFrac metric for microbial communities (Adonis, *p* < 0.01). The NMDS diagrams were constructed using a Bray–Curtis distance matrix for microbial communities (Adonis, *p* < 0.01). The dotted lines represent x = 0 and y = 0. Red circles depict the 95% confidence intervals for the DNA group, while Blue circles represent the 95% confidence intervals for the RNA group.

PCoA results revealed distinct clustering of microbial samples detected by the two methods (DNA vs. RNA, Figure 2A), permutation number = 999, R^2^ = 0.4880, *p* < 0.01). This distinction was further highlighted in the NMDS analysis (DNA vs. RNA, Figure 2B), permutation number = 999, R^2^ = 0.5728, stress = 0.028, *p* < 0.01). A hierarchical clustering analysis showed that the DNA and RNA groups exhibited distinct clustering patterns (Figure 2C). Collectively, NMDS and PCoA revealed distinct clustering (Adonis, R^2^ = 0.4880, *p* < 0.01), indicating RNA-dependent sequencing systematically alters perceived community structure.

### 3.2. Different Bacterial Species Composition and Genus Abundance in the DNA- and RNA-Dependent Sequence Samples

**Figure 3:**
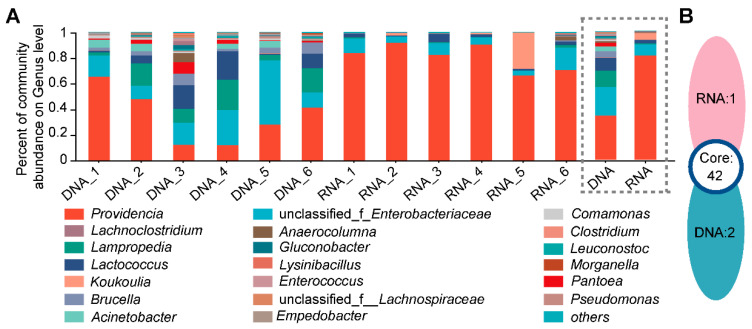
Comparison of species composition of intestinal bacterial microbiota from *D. antiqua* larvae using DNA- and RNA-dependent sequence methods. (**A**) A community stack plot was constructed at the genus level for the intestinal bacterial microbiota obtained from *D. antiqua* larvae. (**B**) presents a Venn diagram illustrating the intestinal bacterial microbiota of *D. antiqua* larvae using DNA- and RNA-dependent sequence methods at the ASV level. The DNA and RNA groups in the dashed box were plotted from the average of all six replicates.

For both the DNA- and RNA-dependent sequencing samples, the dominant bacteria in the intestinal bacterial community were the same, but their proportions in the two groups were different. These bacteria included *Providencia* (DNA: 34.25% vs. RNA: 80.75%), *Lampropedia* (DNA: 12.30% vs. RNA: 0.68%), *Lactococcus* (DNA: 10.04% vs. RNA: 2.72%), *Koukoulia* (DNA: 0.15% vs. RNA: 5.18%), and *Brucella* (DNA: 4.96% vs. RNA: 0.33%) (Figure 3A). At the ASV level (Figure 3B), among these groups, 42 ASVs were present in both groups, with 2 ASVs unique to the DNA group and 1 ASV unique to the RNA group.

**Figure 4:**
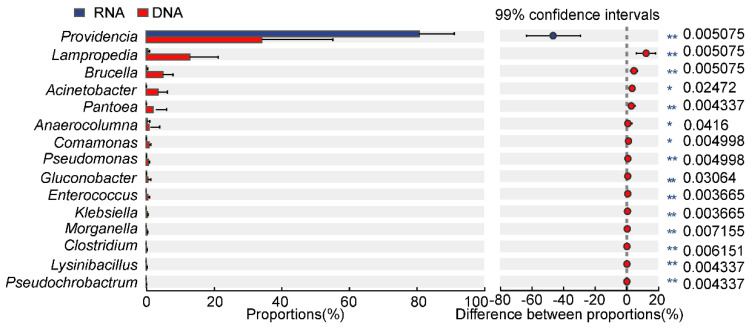
Comparison of the top 15 genus abundance in intestinal bacterial microbiota from *D. antiqua* larvae using DNA- and RNA-dependent sequence methods. The DNA group represents samples tested using DNA-dependent sequence methods, while the RNA group represents those tested using cDNA. The numbers at the right of the figure represent *p*-values from the Mann–Whitney test for the two groups: “*” indicates *p* < 0.05, and “**” indicates *p* < 0.01.

In the RNA group, the abundances of genus *Providencia* were significantly higher than those in the DNA group (Figure 4). For the other remaining 14 genera, their abundances in the DNA group were all higher than those in the RNA group. Significant differences were observed for *Lampropedia*, *Brucella*, *Acinetobacter*, *Pantoea*, *Achromobacter*, *Comamonas,* and the other 8 genera (*p* < 0.05).

### 3.3. SynComs Constructed According to RNA-Dependent 16S rRNA Amplicon Sequencing Showed Inhibition Effects on D. antiqua Larvae

**Figure 5:**
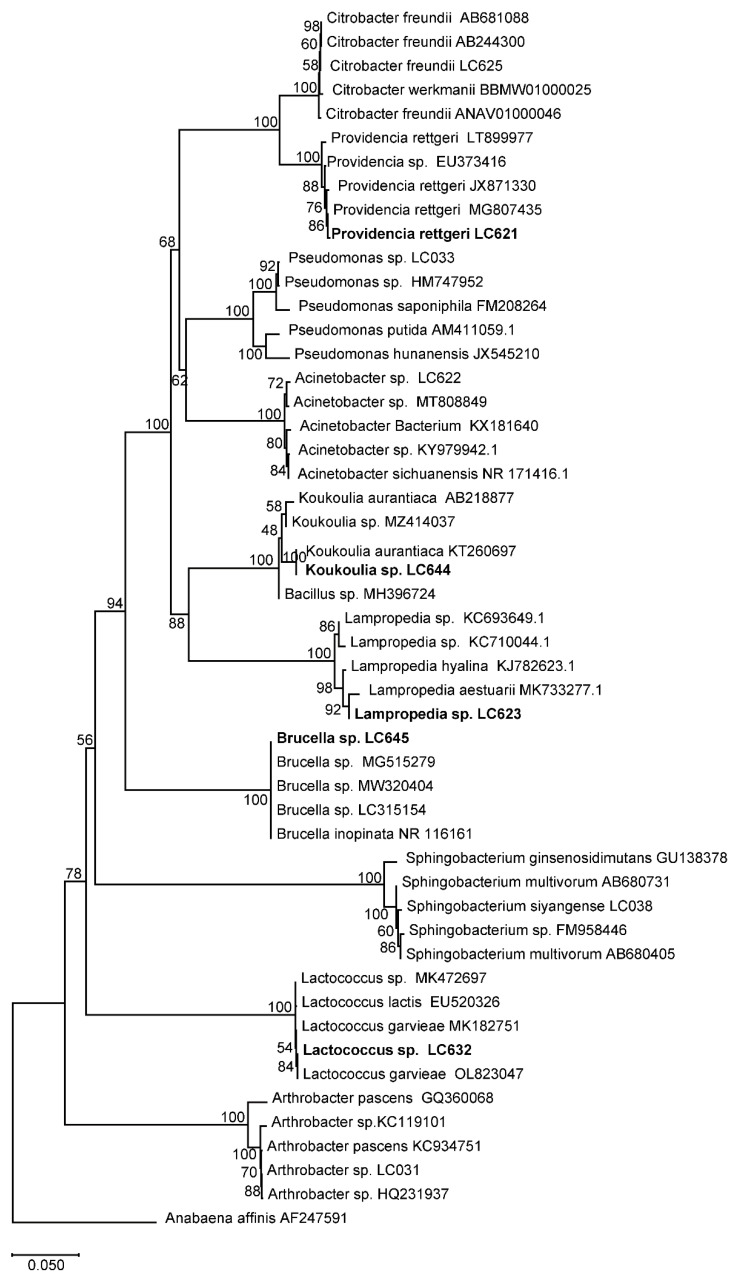
Maximum likelihood tree of bacterial isolates associated with *D. antiqua*. The sequences were obtained through high-throughput sequencing, Numbers on the nodes represent bootstrap support from 1000 replicates. The strains labeled in black font will be used for subsequent experiments.

In total, 330 bacterial strains were isolated from the gut samples of third-instar *D. antiqua* larvae. Those bacterial stains were assigned to 25 bacterial species, 17 genera within 4 phyla (Figure 5). Among those species, bacterial strains including *Providencia rettgeri* LC621, *Lampropedia* sp. LC623, *Lactococcus* sp. LC632, *Koukoulia* sp. LC644, and *Brucella* sp. LC645, belonging to the dominant genera (Figure 4), was selected for further tests.

**Figure 6:**
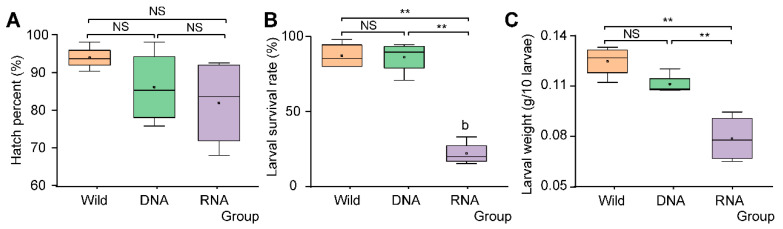
Differences in hatch percent (**A**), larval survival (**B**), and Larval weight (**C**) among various groups of onion flies treated by different SynComs. Each group is represented by a distinct color. A: body weight of 10 larvae. B: larval survival. C: Pupal weight of 10 pupae. D: pupation rate (%). Different letters above boxes denote significant differences (one-way ANOVA followed by Duncan’s multiple tests, *p* < 0.05). “NS” indicates no significant difference, and “**” indicates a significant difference.

SynComs constructed according to RNA/DNA-dependent 16S rRNA amplicon sequencing (RNA-dependent SynCom and DNA-dependent SynCom) and bacterial community from wild *D. antiqua* larval gut samples (Wild community) showed different effects on *D. antiqua* larvae. Specifically, RNA SynCom and DNA SynCom showed no significant effects on egg hatch percentage compared to the Wild community (Figure 6A; one-way ANOVA; F = 1.738; df = 2.9; *p* = 0.230). Differently, larval survival was significantly reduced to 21.24% by RNA-dependent SynCom compared to that of Wild community (Figure 6B; 86.84%; one-way ANOVA; F = 66.407, df = 2.9; *p* < 0.01). However, DNA-dependent SynCom showed no effects on larval survival compared to that of Wild community (Figure 6B; one-way ANOVA; F = 66.407, df = 2.9; *p* = 0.878). The survival of axenic larvae was 66%, and there was a significant difference compared with the RNA-dependent synthetic community (Figure S3A; Independent samples *t*-test; t = − 11.417; *p* < 0.01). Furthermore, the body weight of the larvae (RNA-dependent SynCom) was significantly reduced to 0.0826 g/10 larvae compared to that of the Wild community (Figure 6C; 0.1250 g/10 larvae; one-way ANOVA; F = 20.612, df = 2.9; *p* < 0.01). On the contrary, larval body weight (DNA-dependent SynCom) was not significantly reduced compared to that of the Wild community (Figure 6C; one-way ANOVA; F = 20.612, df = 2.9; *p* = 0.097), In addition, there is no significant difference in the body weight of axenic larvae compared with the RNA-dependent synthetic community (Figure S3B; Independent samples *t*-test; t = 1.029; *p* = 0.343). Further culture-dependent methods revealed that the community from the surviving larval gut was similar to that of SynComs inoculated to sterilized eggs (Supplementary Materials, Figure S2).

## 4. Discussion

This study employed multiple methodologies to assess the diversity of gut bacteria within the pest, *D. antiqua*. Due to the stability of DNA, DNA-dependent detection methods were widely used in pest microbial diversity investigation and exhibit high accuracy [27]. However, these methods fail to distinguish between the alive and dead microbes. Emerging RNA-dependent sequencing techniques can identify alive bacteria by reflecting their activity or transcriptional state, despite the comparatively shorter half-life of RNA relative to DNA [12,13,14,28,29]. This study revealed differences in α-diversity (Figure 1) between the bacterial community of *D. antiqua* obtained using RNA- and DNA-dependent sequencing technology. Additionally, a β-diversity analysis indicated that the microorganisms identified by the two methods exhibited distinct clustering characteristics (Figure 2). Furthermore, significant differences in bacterial abundance between the DNA- and RNA-dependent sequence samples were detected (Figure 4). As more than 97% of the *D. antiqua* gut microbe was bacteria, which was revealed by metagenomic investigation (data unpublished), the gut microbial community of wild larvae was used to inoculate sterilized eggs to represent the full function of the gut bacteria [30]. The SynCom constructed according to RNA-dependent 16S rRNA amplicon sequencing showed inhibition effects on *D. antiqua* larvae compared to that of the gut microbial community of wild larvae, while the SynCom constructed according to DNA sequence did not (Figure 6). In addition, the gut bacterial community of survived larvae was almost the same as the re-inoculated bacterial community in the sterilized eggs (Figure S2). These results indirectly reflected the unreliability of the RNA-dependent amplicon sequence compared to the DNA-dependent sequence in determining the gut bacterial diversity of *D. antiqua*. This work presents the first combination of RNA and DNA sequencing technologies with SynCom re-inoculation assays to compare the reliability of different methods in detecting gut bacterial diversity in *D. antiqua*. It also provides experimental evidence for the unreliability of the RNA-dependent 16S rRNA amplicon sequence in detecting insect gut bacterial diversity.

One essential question for this study was how the bacterial diversity differed between the community obtained through RNA-dependent sequencing and that obtained through DNA-dependent sequencing. Several factors might lead to this difference. On the one hand, several factors might lead to this difference including the relative stability of DNA compared to RNA during extraction and amplification. Biochemical factors such as moisture level, pH, UV exposure, and the presence of nucleases can affect the longevity of rRNA molecules [31,32,33], which might lead to limited species detection. However, the stable DNA might lead to relatively more microbial species, including both the alive microbes and the dead ones in the amplicon sequence [34]. On the other hand, during the preparation of PCR templates for sequencing, the ease or difficulty of extracting DNA and RNA from different species may lead to differences in the species composition of sequencing templates. In addition, the extraction efficiency of DNA is significantly higher than that of RNA [35]. Therefore, during the extraction process, the degradation of RNA may lead to even lower abundance or even failure to be detected for low-abundance species.

Another interesting phenomenon in this study was that some taxa could not be detected by the DNA- or RNA-dependent amplicon sequence, as shown in Figure 3B. This might result from several factors. Firstly, sampling effects can misrepresent the differences between RNA and DNA libraries, especially for rare taxa that can only be detected in larger read libraries due to sampling stochasticity [34]. Secondly, the relative abundances of rare taxa are affected both biologically and technically, and changes in DNA abundances can also contribute to the detection of “phantom taxa” [34]. The polymerase chain reaction (PCR) technology itself has certain limitations. For instance, due to the excessive number of bacterial repetitive sequences and poor primer specificity, primer mismatches may occur [36]. Alternatively, primer bias may also lead to data deviation [37]. The transcription level of the 16S rRNA gene varies with factors such as growth rate, life stages, and exposure to stressors. This can lead to differences in the detection of taxa depending on their growth phase. Due to the degradation characteristics of RNA, the RNA of bacteria present at low abundance is more difficult to detect, resulting in a higher abundance of the dominant bacterium *Providencia*.

Until now, the gut microbial diversity of insects has only been investigated in several species, such as *Prionplus reticularis* [38] and *Spodoptera exigua* using RNA-dependent sequencing [39]. This paper is the first to compare the methods of DNA- and RNA-dependent amplicon sequencing in monitoring the diversity of gut bacteria in *D. antiqua*. However, it does have certain limitations. While 16S-RNA-seq has been employed for assessing microbial community activity, the reliability of this technique requires further testing in different experimental systems, particularly in complex insect–microbe symbiosis, such as in *Plagiodera versicolora* [40], *Bactrocera dorsalis* [41], and *Dendroctonus valens* LeConte [42]. Different environmental conditions have diverse impacts on the experimental procedures. For instance, the yields of DNA/RNA may not be directly comparable after extraction, the reverse transcription efficiency of RNA molecules may vary, and library construction may differ in various environments. These technical factors will also affect the outcomes of 16S-RNA-seq [7]. Therefore, in future practical applications, attention should be paid to multi-omics integration, combining 16S-RNA-seq with functional indicators (such as metatranscriptomics or metaproteomics profiles) to mutually verify the detection results [34]. It would be helpful to conduct qPCR tests to quantify the abundance of each genus to confirm the reliability of the DNA- or RNA-dependent amplicon sequence. However, this was not conducted in this work as the microbial community was too complex to select specific primers for each genus/species.

## 5. Conclusions

In conclusion, this study focused on the global pest *D. antiqua* and utilized multiple methodologies to assess its associated bacterial diversity. The study found differences in α-diversity and β-diversity between bacterial communities obtained using RNA- and DNA-dependent amplicon sequencing. Significant differences in bacterial genus abundance were also detected.

SynCom re-inoculation tests revealed the unreliability of RNA-dependent 16S rRNA amplicon sequence in detecting insect gut bacterial diversity.

## Data Availability

The datasets generated during and analyzed during the current study are available from the Genome Sequence Archive (Genomics, Proteomics & Bioinformatics 2021) in National Genomics Data Center (Nucleic Acids Res 2022), China National Center for Bioinformation/Beijing Institute of Genomics, Chinese Academy of Sciences (GSA: CRA019195) that are publicly accessible at https://ngdc.cncb.ac.cn/gsa (accessed on 30 October 2024).

## Supplementary Materials

The following supporting information can be downloaded at: https://www.mdpi.com/article/10.3390/insects16060611/s1. Supplementary Methods: Detailed information on DNA/RNA dependent amplicon sequencing. Supplementary Figure S1: Rarefaction curves of α index for intestinal bacterial microbiota from *D. antiqua* Larvae. Supplementary Figure S2: The proportion of community from the survived larval gut and SynComs inoculated to sterilized egg, including five bacterial species: Providencia rettgeri LC621, Lampropedia sp. LC623, Lactococcus sp. LC632, Koukoulia sp. LC644, and Brucella sp. LC645. Supplementary Figure S3: The differences in (A) larval survival rate and (B) larval weight of *D. antiqua* between the RNA synthetic community treatment and the sterile control group are shown. Each group is represented by a distinct color. Different letters above the boxes indicate significant differences: “NS” denotes no significant difference, and “**” indicates a significant difference (independent samples *t*-test, *p* < 0.05).
