# Supplementary material for: Laboratory Test Indirectly Reveals the Unreliability of RNA-Dependent 16S rRNA Amplicon Sequences in Detecting the Gut Bacterial Diversity of Delia antiqua
Source: Insects. 2025 Jun 10;16(6):611. doi: 10.3390/insects16060611 (PMC12193304; doi:10.3390/insects16060611)
Supplement: Supplementary file 1 [file insects-16-00611-s001.zip › insects-3668398-supplementary.pdf]

Laboratory tests indirectly reveal the unreliability of RNA-dependent 16S rRNA amplicon sequence in detecting gut bacterial diversity of *Delia antiqua*

Running title: RNA-dependent amplicon sequence is unreliable

Miaomiao Li[1], Xin Cao[1], Linfeng Xu[1], Luyao Lin[1], Xiaoqing Wu[1], Susu Fan[1], Xinjian Zhang[1*], Fangyuan Zhou[1*]

[1] Shandong Provincial Key Laboratory of Applied Microbiology, Ecology Institute, Qilu University of Technology (Shandong Academy of Sciences), Ji'nan, 250103, P.R. China.

*Correspondence: Xinjian Zhang (zhangxj@qlu.edu.cn), Fangyuan Zhou (fangyuan_zhou@qlu.edu.cn), Ecology Institute, Qilu University of Technology (Shandong Academy of Sciences), No. 28789 Jingshidong Road, Licheng District, Ji'nan, 250103, China. Tel: 86-531-8872 8703.

Simple Summary

The symbiotic relationship between insects and microorganisms plays a crucial role in the fitness and ecological adaptation of insects. As one of the most threatening pests to Liliaceae crops worldwide, the Delia antiqua has been thoroughly studied in terms of its biological characteristics and microbial symbionts. We utilized DNA-dependent and RNA-dependent 16S rRNA amplicon sequencing to assess the gut bacterial diversity of D. antiqua. The results showed that RNA-dependent sequencing is unreliable for detecting gut bacterial diversity. These findings are of great significance for selecting reliable methods to study the bacterial diversity in the symbiotic relationship between insects and microorganisms.

Supplementary Methods

Detailed information on DNA/RNA dependent amplicon sequencing

The gene was amplified using primer pairs 338F (5'-ACTCCTACGGGAGGCAGCAG-3') and 806R (5'-GGACTACHVGGGTWTCTAAT-3') with an ABI GeneAmp® 9700 PCR thermocycler (ABI, CA, USA). The PCR amplification of the 16S rRNA gene was carried out as follows: initial denaturation at 95°C for 3 min, followed by 27 cycles of denaturation at 95°C for 30 seconds, annealing at 55°C for 30 seconds, extension at 72°C for 45 seconds, and a final extension at 72°C for 10 min, with a final step at 4°C. The PCR mixtures consisted of 4 μL of 5 × TransStart FastPfu buffer, 2 μL of 2.5 mM dNTPs, 0.8 μL of forward primer (5 μM), 0.8 μL of reverse primer (5 μM), 0.4 μL of TransStart FastPfu DNA Polymerase, 10 ng of template DNA, and finally, add ddH_2O up to 20 μL. PCR reactions were conducted in triplicate, and the products were mixed for further sequencing. The PCR product was purified using the AxyPrep DNA Gel Extraction Kit (Axygen Biosciences, Union City, CA, USA) following the manufacturer's protocol, and quantified using a Quantus™ Fluorometer (Promega, USA).

The raw sequencing reads of the 16S rRNA gene were demultiplexed and quality-filtered using fastp version 0.20.0 [1]. The filtered reads were then merged using FLASH version 1.2.7 [2]. The filtering criteria included truncating reads of 300 bp length at sites with an average quality score <20 within a 50 bp sliding window. Truncated reads shorter than 50 bp and reads containing ambiguous characters were discarded. Overlapping sequences longer than 10 bp were assembled based on their overlap, allowing a maximum mismatch ratio of 0.2 in the

overlap region. Reads that could not be assembled were discarded. Sample discrimination was performed by matching barcodes and primers, with the sequence direction adjusted to ensure exact barcode matching and allowing for 2 nucleotide mismatches in primer matching. Operational taxonomic units (ASVs) were clustered at a 97% similarity cutoff using UPARSE version 7.1. Chimeric[3] sequences were detected and eliminated. The taxonomic classification of each representative sequence of an ASV was performed using RDP Classifier version 2.2 [4] with a confidence threshold of 0.7 against the 16S rRNA database (e.g., Silva v138). Taxa were then filtered at thresholds of > 0.01% relative abundance in at least 10% of all samples.

The calculations for Figures 1-4 were performed using tools on an online cloud platform. Alpha diversity was statistically tested using R-3.3.1 (stat). For beta diversity, Venn diagrams were created using Python 2.7.10, Community barplot analysis was statistically tested using Python 2.7, and Wilcoxon rank-sum test bar plots were tested using R-3.3.1 (stat). Figure 6 was statistically analyzed and tested using SPSS 26.0 software.

Supplementary Figures

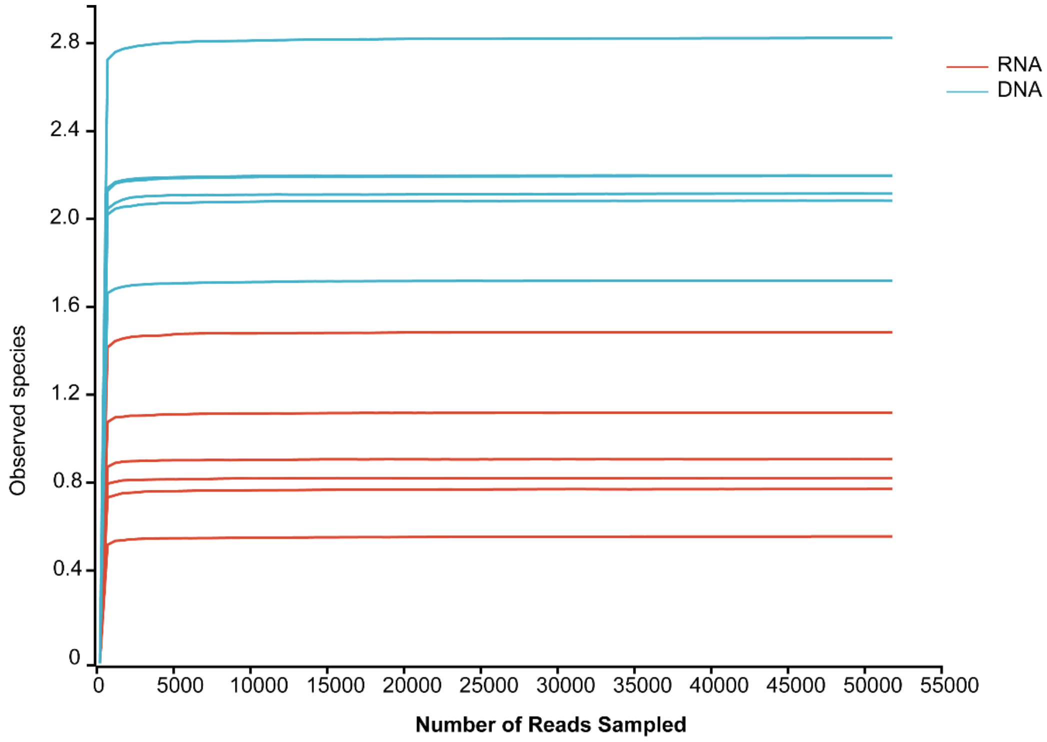

Figure S1 Rarefaction curves of α index for intestinal bacterial microbiota from *D. antiqua* larvae

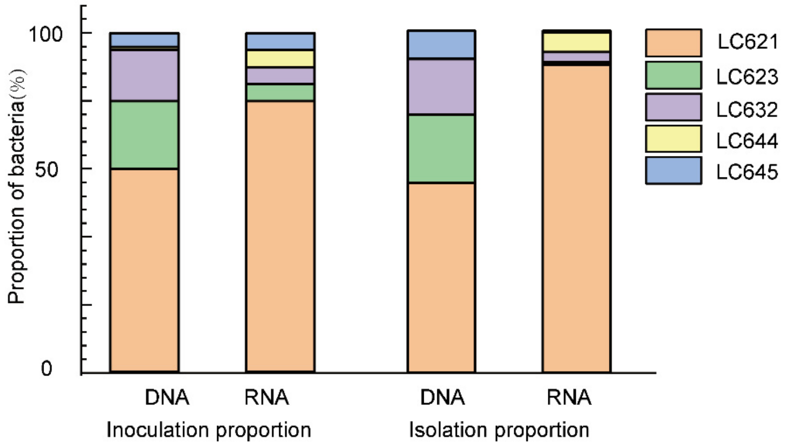

Figure S2: The proportion of community from the survived larval gut and SynComs inoculated to sterilized egg, including five bacterial species: Providencia rettgeri LC621, Lampropedia sp. LC623, Lacto-coccus sp. LC632, Koukoulia sp. LC644, and Brucella sp. LC645.

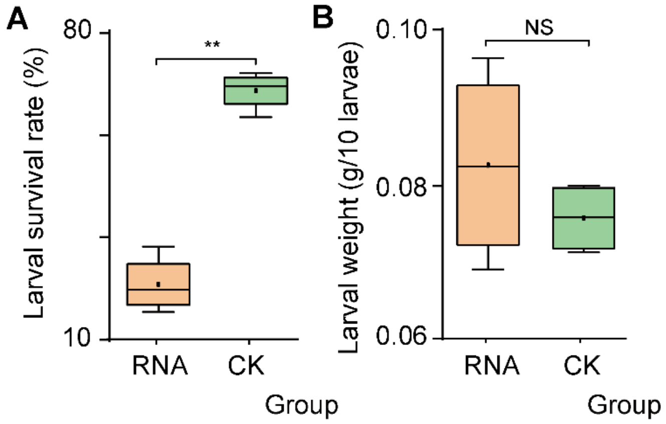

Figure S3: The differences in (A) larval survival rate and (B) larval weight of D. antiqua between the RNA synthetic community treatment and the sterile control group are shown. Each group is rep-resented by a distinct color. Different letters above the boxes indicate significant differences: "NS" denotes no significant difference, and "**" indicates a significant difference (independent samples t-test, $p < 0.05$).

References

1. Chen, S.; Zhou, Y.; Chen, Y.; Gu, J. fastp: an ultra-fast all-in-one FASTQ preprocessor. *Bioinformatics* **2018**, *34*, 884-890, doi:https://doi.org/10.1093/bioinformatics/bty560.
2. Tanja, M.; L, S.S. FLASH: fast length adjustment of short reads to improve genome assemblies. *Bioinformatics (Oxford, England)* **2011**, *27*, 2957-2963, doi:https://doi.org/10.1093/bioinformatics/btr507.

3. Stackebrandt, E.; Goebel, B.M. Taxonomic note: A place for DNA:DNA reassociation and 16s rRNA sequence analysis in the present spec. *International Journal of Systematic Bacteriology* **1994**, *44*, 846-849, doi:https://doi.org/10.1099/00207713-44-4-846.
4. Wang, Q.; Garrity, G.M.; Tiedje, J.M.; Cole, J.R. Naive Bayesian classifier for rapid assignment of rRNA sequences into the new bacterial taxonomy. *Applied and Environmental Microbiology* **2007**, *73*, 5261-5267, doi:https://doi.org/10.1128/aem.00062-07.
